# Doxorubicin-loaded red blood cells reduced cardiac toxicity and preserved anticancer activity

**DOI:** 10.1080/10717544.2019.1591544

**Published:** 2019-03-31

**Authors:** Alfredo Lucas, Dawn Lam, Pedro Cabrales

**Affiliations:** Department of Bioengineering, University of California, San Diego, CA, USA

**Keywords:** Drug delivery, doxorubicin, biomimetic, red blood cell

## Abstract

Doxorubicin (DOX) is one of the most widely used anticancer agents. DOX is known for inducing cardiotoxicity, resulting in the long-term development of heart failure. Intravascular delivery of DOX may benefit from the carriage by red blood cells (RBCs), as they can limit the systemic toxicity while delivering the DOX to the tumor. This study proposes a methodology for the synthesis of electrophoretically DOX-loaded red blood cells (RBC-DOX), as well as the assessment of its antitumorigenic effects in human colon cancer cells (HT-29), and in colon cancer xenograft models. In addition, healthy mice without tumors were dosed with RBC-DOX to assess cardiotoxicity via assessment of indexes of cardiac function after multiple doses of RBC-DOX. The HT-29 IC_50_ was found to be lower for RBC-DOX compared to free DOX. Tumor volume for the RBC-DOX group was smaller than the free DOX groups in HT-29 xenografts models. Statistically higher concentrations of DOX were found in the liver, spleen, and lungs for the RBC-DOX group compared to the free DOX group. However, the heart and the skin had statistically lower DOX concentrations for the RBC-DOX group compared to the free DOX group, with no significant differences in tumor biodistribution. All hemodynamic and cardiac function parameters were closer to control parameters for the RBC-DOX treated compared to for the free DOX-treated mice. These results suggest that RBC-DOX can be an alternative to prolong treatments with DOX, with superior antitumorigenic effects, decreased myelosuppression, and limited cardiac toxicity compared to equivalent doses of free DOX.

## Introduction

Colorectal cancer is the third most common cause of cancer-associated deaths (Jemal et al., 2010), with a high percentage of subjects presenting metastatic disease. Despite surgery and chemotherapy, subjects with colorectal cancer eventually succumb to metastatic diseases. The success of radiotherapy and chemotherapy in colorectal cancer is limited by the resistant cancer cells and the dose-limiting toxicities (Segal & Saltz, 2009). Anthracyclines, are considered some of the most effective anticancer drugs available in the market (Weiss, 1992). Doxorubicin (DOX) is one of the most common anthracyclines, been proven effective against soft tissue and bone sarcomas, and breast, ovary, bladder, and thyroid cancer. It has also been used for the treatment of small cell lung cancer, Hodgkin’s lymphoma, and acute myeloblastic and lymphoblastic leukemia (Johnson-Arbor & Dubey, 2018). However, anthracyclines have well-known side effects, where amongst the most common is cardiotoxicity (Chatterjee et al., 2010; Mitry & Edwards, 2016). Doxorubicin cardiomyopathy is frequently fatal once developed, as it often evolves into a form of congestive heart failure unresponsive to treatment (Bristow et al., 1978; Takemura & Fujiwara, 2007). In a large-scale retrospective cohort study, older women diagnosed with breast cancer were followed for a period of 9 years, and the leading cause of death was cardiovascular disease, followed by breast cancer itself (Patnaik et al., 2011). Although the severity of the cardiomyopathy is dose-dependent (Alexander et al., 1979), so is the drug’s effectiveness, therefore, methods to decrease the exposure of noncancerous tissue to DOX, while still maintaining a sufficient tumor DOX exposure.

An approach that has been in the spotlight since the early 80 s has been the use of liposomes for anticancer drug delivery, including the delivery of DOX (Gregoriadis, 1985). Liposomes are nanoscale-sized spheroids with a lipid-based membrane, which allow the encapsulation of substances as a means of drug delivery. By enclosing the delivered substance, they allow for a longer circulation time, and therefore an increased opportunity for the drug to arrive to the region of interest. Early efforts in the use of liposomes for the delivery of DOX, however, were thwarted by the early recognition of the liposomes by the reticulo-endothelial system, resulting in fast degradation and plasma clearance (Juliano & Stamp, 1975; Poste 1982). A great body of research has focused on the modification of the surface properties of the liposomes in order to prolong their circulation time and efficacy. One line of research has focused in adding bulky synthetic groups such as polyethylene-glycol (PEG) to the surface of DOX-loaded liposomes, allowing for decreased opsonization, and therefore, longer circulation times (Gabizon et al., 2003). However, recent findings relating to PEG-induced immune response have led to the search for alternatives (Knop et al., 2010). Some groups have focused in mimicking surface markers and structural features present in red blood cells (RBCs) and adding them to the liposomes or nanoparticles and have been proven successful (Doshi et al., 2009; Parodi et al., 2013; Li et al., 2014; Luk & Zhang, 2015; Zhai et al., 2017). However, these techniques often involve multi-step procedures, and since there is still a synthetic component, namely the nanoparticle or the liposome, the immune response is still likely to eventually occur and there is a potential for toxic byproducts during degradation. Currently, DOX is clinically administered as Doxil®, a pegylated liposomal doxorubicin. This formulation is preferred above free DOX as it prolongs DOX circulation time and avoids reticuloendothelial system activation by the liposomal lipid bilayer due to the use of PEGylated nano-liposomes. Doxil® has shown less cardiac toxicity than free DOX on an equimolar basis, but in both cases the cardiac risk increases with increased cumulative dose (O’Brien et al., 2004).

Other approaches to mitigate the toxicity of doxorubicin have embedded DOX inside human serum albumin (HSA) aggregates, with or without surface modification to increase tumor specificity (Bae et al., 2012; Zheng et al., 2015). The surface modifications of the HSA aggregates include the amino-terminal fragment of urokinase (ATF), which binds with a high affinity to urokinase receptor overexpressed in many types of tumors, or the tumor necrosis factor (TNF)-related apoptosis-inducing ligand (TRAIL) and transferrin to increase cytotoxic and apoptotic activities (Bae et al., 2012; Zheng et al., 2015). Although, these approaches reduce the cardiotoxicity of DOX and increase antitumor efficacy, HSA based formulations affect plasma protein concentration, plasma colloid osmotic pressure, and hematocrit pre- and post-infusion. In addition, as HSA is produced by fractionation of plasma obtained from donors, the theoretical potential for the transmission of new and reemerging infectious agents (hepatitis, human immunodeficiency virus, West Nile virus, etc) with blood‐ and plasma‐derived products is not unlikely to be eliminated. On the other hand, the use of RBCs as a bio-compatible, liposome-like, drug delivery alternative has been explored extensively (Muzykantov, 2010; Xu et al., 2016; Sun et al., 2017). The advantage of using RBCs, as opposed to traditional coated liposomes or nanoparticles, lies in their superior biocompatibility and absence of toxic byproducts during degradation, especially when autologous blood types are used (Pierigè et al., 2008). These advantages come while still preserving the long circulation times and sustained release ability present in the other drug delivery approaches (Sun et al., 2017). Furthermore, removing the need to coat the nanoparticle or liposome, the synthesis process can be simplified, increasing efficiency. Additionally, their specific use for the delivery of DOX has been successfully demonstrated previously by two studies, one in humans for the treatment of lymphoma (Ataullakhanov et al., 1997; Skorokhod et al., 2007), and one in dogs for the treatment of lymphosarcoma (Matherne et al., 1994), proving successful in both cases. Neither of these studies, however, performed an in-depth analysis of the cardiotoxicity derived from these loaded cells. Additionally, they did not show any results pertaining to the biodistribution, myelosuppression, immune response, and effective cytotoxicity resulting from the use of doxorubicin-loaded red blood cells (RBC-DOX), all of which are necessary for a proper understanding of the potential benefits, and drawbacks, of using RBC-DOX as a delivery alternative for DOX. We have recently developed a novel approach to load drugs into red blood cells to use them as biological carrier systems (Cabrales et al., 2008; Villela et al., 2009).

We have also demonstrated that the electrophoretically modified RBCs do not lose hemoglobin (Hb) through hemolysis after infusion and that loading drugs in RBCs does not affect their deformability significantly (Cabrales et al., 2008; Villela et al., 2009). In this study, we propose a methodology for the synthesis of RBC-DOX, as well as the assessment of its antitumorigenic effects *in vitro* in human adenocarcinoma HT-29 cells, and *in vivo* in HT-29 implanted athymic mice. Myelosuppression, biodistribution and tumor growth were all assessed *in vivo* for the HT-29 implanted mice. Healthy mice were also treated with equivalent formulations of both free DOX and RBC-DOX, and their systemic hemodynamics and cardiac function parameters were assessed in order to determine cardiac toxicity.

## Methods

### RBC-DOX preparation

Blood was collected from mice donor (25–30 g) into a vacutainer (EDTA). Red cells (RBCs) were isolated and washed three times by centrifugation. Electroporation was used to load doxorubicin (DOX) into RBCs. A high voltage pulse generator BTX T100 (Biotechnologies and Experimental Research, San Diego, CA) was used to create micropores to allow slow diffusion of DOX from medium into the RBCs. BTX T100 was configured to deliver repeated pulses (3.5 kV/cm followed by an exponential decay, tau = 6 sec) for 3 mins. The electroporation chamber was a modified sterile cuvette (0.5 mL) with parallel stainless steel electrodes separated 2.2 mm gap. RBCs (at 50% Hct) were suspended in saline solution with DOX at 5 mg/mL. RBCs were resealed by incubation at 4 °C (5 min) followed by 37 °C (1 h). Lastly, RBC-DOX were then washed twice with PBS with 0.5% HSA.

### DOX loading efficiency

Doxorubicin loading amount and efficiency were calculated indirectly by subtracting the measured amount of drug in washed solution from the initially added drug amount, and directly after hemolyzing RBCs in methanol. Doxorubicin in the collected wash solution was analyzed by high-performance liquid chromatography (HPLC) (Urva et al., 2009). The HPLC system used was a Hitachi D-7000 series (Hitachi, Japan); components consisted of a Masterflex L/S Economy pump (Barnant Company, USA), Hitachi L-7485 fluorescence detector with excitation and emission wavelength set at 480 and 560 nm, respectively. A mixture of methanol and 10 mM phosphate buffer (pH = 3.0) was used as the mobile phase. The flow-rate used in the assay was 0.8 mL/min and the column was maintained at 40 °C throughout the chromatographic process. The loading efficiency curve is shown in Supplemental Figure 1.

**Figure 1.**
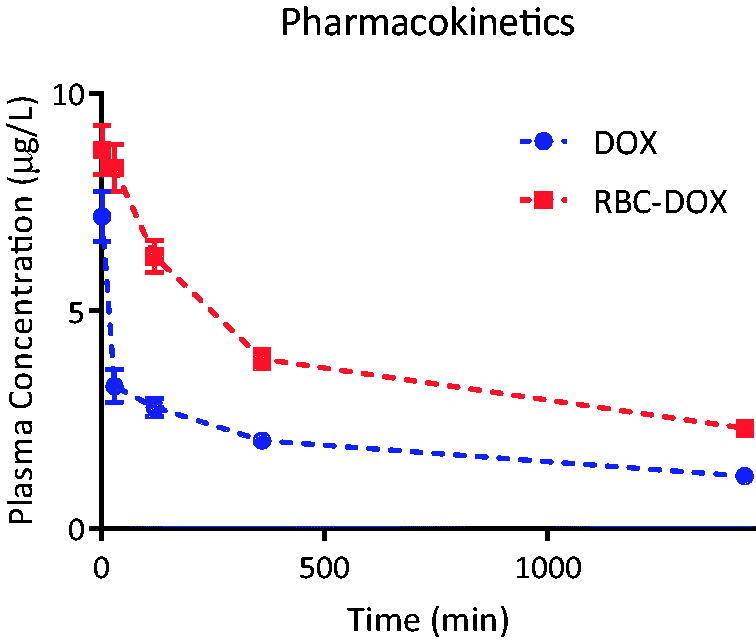
Plasma concentration of DOX (blue), and RBC-DOX (red), as a function of time over a 24 h time period after a 5 mg/kg intravenous injection. Corresponding area under the curves and plasma clearance rate are shown in Table 1.

### Colorectal cytotoxicity of DOX-RBC

Human colorectal adenocarcinoma HT-29 cells (ATCC, Manassas, VA) were cultured at 37 °C in 95%O_2_, 5% CO_2_. The cultures were randomized into two groups, DOX and RBC-DOX, of *n* = 8 wells each. Each well contained 2 × 10^3^ cells in McCoy’s 5 A medium with 10% FBS. In the DOX group, DOX solution ranging in concentration between 0 and 3 µg/mL was added to each well 24 h after cell plating. Medium was replaced every 24 h with DOX-RBC or DOX equivalent concentration ranging between 0 and 3 µg/mL, as dictated by the loading efficiency curve. After 48 h of incubation, absorbance was measured, and cytotoxicity was expressed as a percentage of control cells, which received no treatment. The inhibition concentration 50% (IC50), defined as the dose of agents that inhibited 50% of cell growth, was interpolated from the growth curves. All experiments were performed in triplicate and repeated several times.

### Pharmacokinetics

Tumorless 6–7 weeks old mice (C57BL, 20–25g) were administered a single intravenous (IV) dose of DOX (*n* = 3) or RBC-DOX (*n* = 3) at 5 mg DOX/kg. Blood samples were collected in heparinized tubes from the tail at 2 min, 0.5 h, 2 h, 6 h, and 24 h. After collection, blood samples were centrifuged to separate RBCs and plasma. To determine DOX levels in plasma, 200 µl of methanol and 200 µl of phosphate buffer were then added to 25 µL plasma, vortexed for 1 min, and centrifuged again. The supernatant was mixed with 1 mL of perchloric acid (35%, v/v) and vortexed for 1 min, and centrifuged one more time prior measurement of DOX concentration using HPLC.

### Experimental groups

Groups were labeled based on the treatment given, namely: DOX, for the animals that received free DOX, and RBC-DOX, for animals that received DOX-loaded RBCs. Where appropriate, control animals were included and were either left untreated or received saline vehicle solution.

### *In vivo* experimental model

Xenograft tumors were established in 6 weeks old female athymic nude (nu/nu) mice, obtained from Jackson Labs (Bar Harbor, ME) and maintained under pathogen limited conditions. All animal experiments were performed in accordance with the guidelines of Institutional Animal Welfare Committee. Animals were fed *ad libitum* with a standard diet. Tumor cells growing exponentially were harvested by brief incubation with 0.25% trypsin EDTA solution. The cells were washed and resuspended at a concentration of 3 × 10^7^ cells/mL in PBS, which was then inoculated subcutaneously (s. c.) into the right flank of the mice. Tumor size was assessed using a digital caliper every other day after implantation and approximate tumor volume (mm^3^) was calculated as length × width^2^/2 (V = lw^2^/2). Treatment started when tumors were 150 mm^3^. Mice were randomized into three groups DOX, RBC-DOX, and control untreated (*n* = 6 per group). The DOX received 5 mg/kg IV doses twice a week for 4 weeks. The RBC-DOX group received an equivalent 5 mg/kg IV doses twice a week for 4 weeks. The control group received no treatment.

### DOX biodistribution

Studies comparing the accumulation of free DOX or RBC-DOX in tumors and organs (liver, spleen, lungs, kidney, heart, and skin) were performed in the HT-29 tumor xenograft model. Tissues and tumor were assessed through HPLC after tissue homogenization after 4 weeks (24 days) of treatment.

### Analysis of myelosuppression

To evaluate the general myelosuppressive activity of DOX and RBC-DOX, whole blood was collected into tubes with EDTA when the mice for therapeutic experiments were sacrificed. Blood cells counts were analyzed using a Hemavet 950FS Multi-Species Hematology System (Drew Scientific, CT) programed with mouse settings. Additionally, blood smears were obtained for each animal to obtain a relative white cell count. Slides were stained with Giesma. The total number of white cells per 1000 red cells were counted (*n* = 3 for each slide).

### DOX-induced cardiotoxicity model

BALB/c mice weighing 22–25 g (Jackson Laboratories, Bar Harbor, ME) were separated into DOX, RBC-DOX, and control groups (*n* = 6 per group). The DOX group received 5 mg/kg IV doses of DOX, the RBC-DOX group received 5 mg/kg equivalent of DOX and the control group received a vehicle infusion of saline. The animals were treated at days 0, 3, 6, and 9. Hemodynamic measurements of heart rate (HR), mean arterial pressure (MAP), and systemic vascular resistance (SVR) were completed 5 days after final administration. Animals were anesthetized using sodium pentobarbital (40 mg/kg IP). Animal preparation included: (i) left femoral artery catheterization, (ii) tracheotomy (polyethylene-90 tube), and (iii) left ventricle conductance catheter introduction through the right carotid artery. Animals were placed in the supine position on a heating pad to maintain core body temperature at 37 °C. Animals were mechanically ventilated (TOPO ventilator, Kent Scientific, CT) using room air (respiration rate of 90 breaths per minute; peak inspiratory pressure of 20 cmH_2_O). After instrumentation, volatile anesthesia (0.6%/vol Isoflurane, Drägerwerk AG Lübeck) was administered using a vaporizer connected to the ventilator. Deep of anesthesia was continually verified via toe pinch, if needed, isoflurane was increased by 0.1%/vol to prevent animal discomfort.

### Cardiac function

The closed chest method was used to study cardiac function. Briefly, the right common carotid artery was exposed to insert a 1.4 F pressure-volume conductance catheter (pressure-volume PV catheter; SPR-839, Millar Instruments; Houston, TX). The PV catheter was advanced passing through the aortic valve into the left ventricle (LV). The pressure and volume signals were continuously acquired (MPVS300, Millar Instruments; Houston, TX and PowerLab 8/30, AD Instruments; Colorado Springs, CO). Left ventricular volume was measured continuously in conductance units (RVU; relative volume unit) and converted to actual blood volume (μL) at the end of the experiment. Parallel volume was calibrated at the end of the experiment via IV injection of 10 µL hypertonic saline (15%). Cardiac function was analyzed with PVAN software (Millar Instruments, TX). Cardiac function parameters were averaged from 10–15 cardiac cycles at each time point. End-systolic pressure (P_es_) was directly measured. Maximum rate of pressure change (dP/dt_max_), minimum rate of pressure change (dP/dt_min_), maximum filling volume rate (dV/dt_max_), ejection fraction (EF), cardiac output (CO), and stroke work (SW) were calculated. Systemic vascular resistance (SVR) was calculated as SVR = MAP/CO.

### Statistical analysis

Results are presented as mean ± standard deviation. As the data were collected, interim analysis was implemented, and following animal care regulation, no more animals were included as statistical significance was reached. Statistically significant changes between solutions and time points were analyzed using two-way analysis of variance (two way ANOVA), followed by *post-hoc* analyses using Tukey's multiple comparisons test when appropriate. All statistics were calculated using GraphPad Prism 6 (GraphPad, San Diego, CA). Results were considered statistically significant if *p* < .05.

## Results

### Characterization of RBC-DOX

Loading of DOX into RBC was found proportional to the volume of RBCs used (2.6 mg/mL of blood). Intracellular retention of DOX during storage was determined by taking advantage of the natural fluorescent property of DOX. The RBC cellular uptake and retention of DOX was studied using fluorescent microscopy. In addition, HT-29 cells uptake of DOX from DOX-RBC was also confirmed via incubation of DOX-RBC and HT-29 cells for 24 h. After washing, the RBC and HT-29 cellular uptake of different RBCs electrophoretically loaded with DOX were evaluated to determine optimal loading conditions and DOX concentrations. The HT-29 cells treated with free DOX showed slightly greater red fluorescence than those treated with RBC-DOX after 24 h incubation. However, the retention of DOX in the RBC-DOX in extended and gradually decreased over 24 h, ensuring the small amounts of DOX were slowly released to HT-29 cells ensuring continuous challenge of cells. The prolonged release and retention of RBC-DOX *in vitro* suggest that RBCs might have superior efficacy and reduced toxicity *in vivo*.

### *In vitro* experimental model

The cytotoxicity of DOX and RBC-DOX in HT-29 cells is shown in Supplemental Figure 2. The IC50 was 1.77 µg/mL for the free DOX group, and 1.45 µg/mL for the RBC-DOX group, as calculated by linear interpolation between the points around 50%. At the maximum concentration of 3 µg/mL the median percent survival was 27.55% for free DOX and 6.65% for RBC-DOX. These results indicate an increased tumor cytotoxicity for RBC-DOX as compared to DOX.

**Figure 2.**
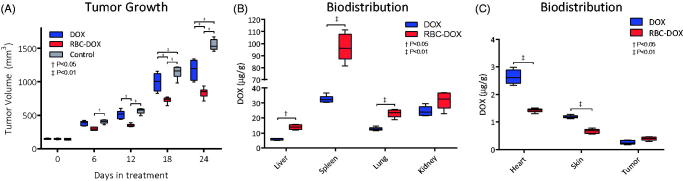
(A) Tumor growth as measured by tumor volume, as a function of time, over a 24 day period for control (gray), DOX (blue) and RBC-DOX (red) treated mice. (B) Biodistribution as measured by post-treatment concentration of DOX in liver, spleen, lungs, and kidney, and (C) heart, skin, and tumor, for DOX (blue) and RBC-DOX (red) treated mice after 24 day treatment period.

### Pharmacokinetics

The DOX plasma concentration as a function of time for DOX and DOX-RBC after a single 5 mg/kg IV dose is shown in Figure 1. Table 1 summarizes the comparisons between the two curves. The area under the concentration curve during the 24 h was statistically higher for RBC-DOX compared to DOX. The same improvement was found in the plasma concentration-time curve. The total body clearance calculated as the exponential decay time constant of the concentration curve was statistically greater and almost double for RBC-DOX compared to DOX. These results indicated a longer lasting presence and enhanced bioavailability of DOX in the body when loaded into RBCs as compared to free DOX.

**Table 1.** Comparison of pharmacokinetics.

Parameter	DOX	RBC-DOX
Dox dose (mg/kg)	5	5
AUC_0–24 h_ (µg/Lh)	1289 ± 48	3024 ± 89
CL (L/h/kg)	2.79 ± 0.31	1.49 + 0.09

Pharmacokinetic from i.v. injection at 5 mg/kg.

Data, means ± SEM; (*n* = 3).

AUC_0–24 h_: Area under plasma concentration; CL: Total body clearance.

### *In vivo* experimental model

Figure 2 summarizes the results of the *in vivo* experimental model. The tumor volume over time for the *in vivo* experimental model for free DOX, RBC-DOX, and control is shown in Figure 2(A). All groups were no different at the beginning of treatment. At all timepoints tumors treated with RBC-DOX have a significantly smaller tumor volume as compared to the untreated control, and after 12 days of treatment significantly smaller tumor volume compared to the free DOX group. The mean final tumor volumes were significantly smaller for RBC-DOX compared to DOX and untreated control. The biodistribution of DOX in the free DOX and RBC-DOX groups is shown in Figure 2(B,C). The liver, spleen, and the lungs of the RBC-DOX group all had a significantly higher concentration of DOX, relative to the free DOX group (Figure 2(B)). On the other hand, the skin and the heart had both lower concentrations of DOX in the RBC-DOX group as compared to the free DOX group (Figure 2(C)). The tumor and the kidneys both had slightly larger concentrations of DOX in the RBC-DOX group compared to the free DOX group. Our data demonstrate that the accumulation of DOX tissues more susceptible to DOX toxicity was significantly lower than that of DOX. Since tissue levels of DOX were reduced by RBC-DOX delivery, the decreased concentration of DOX at these sites is likely to result in a reduced risk of the development of side effects. The increased accumulation of DOX in the liver, spleen, and lung with RBC-DOX compared to the free DOX is the results of RBC loaded cells is expected as these organs have extended capillary networks and are part of the organs of the reticuloendothelial system.

**Figure 3.**
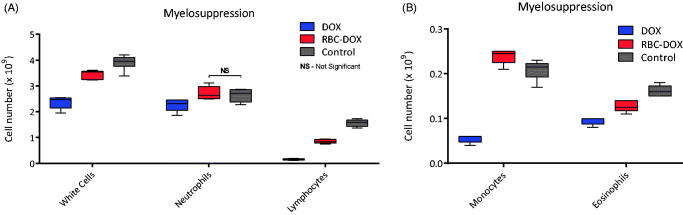
Myelosuppression as measured by cell count of (A) white cells, neutrophils, and lymphocytes, and (B) monocytes and eosinophils, for DOX (blue) and RBC-DOX (red) treated, and control (gray) mice. All differences were statistically significant (*p < .*01), except between RBC-DOX and control neutrophil count.

The myeloid cell counts used to assess myelosuppression are shown in Figure 3. All cell counts were statistically different between each group except for neutrophils when compared between RBC-DOX and control groups, where no statistical difference between cell counts was found. The RBC-DOX group had higher counts for all myeloid cell types than the DOX, and higher monocyte cell count than both control and free DOX group.

### DOX-induced cardiotoxicity model

**Figure 4.**
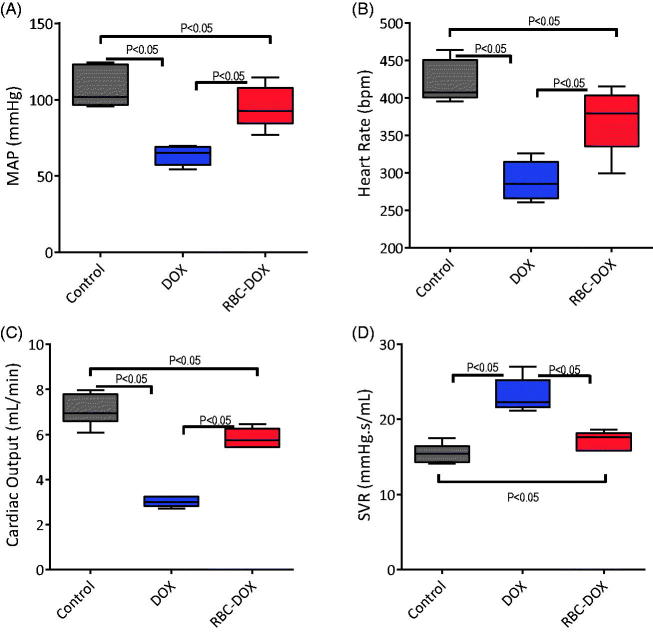
Hemodynamic parameters for control (gray), DOX (blue), and RBC-DOX (red) treated mice. (A) mean arterial pressure (MAP), (B) heart rate, (C) cardiac output (CO), and (D) systemic vascular resistance (SVR).

**Figure 5.**
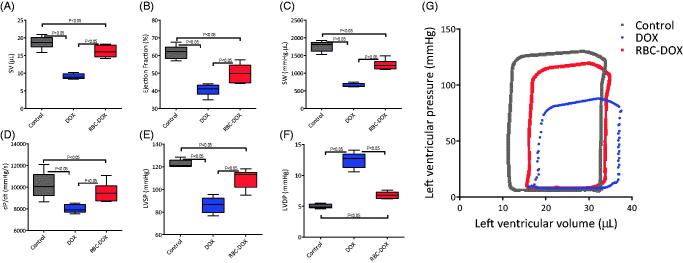
Cardiac function parameters for control (gray), DOX (blue), and RBC-DOX (red) treated mice. (A) stroke volume (SV), (B) ejection fraction (EF), (C) stroke work (SW), (D) contractility (dP/dt), (E) left ventricular systolic pressure (LVSP), and (F) left ventricular diastolic pressure (LVDP). (G) shows the PV-loops from which the cardiac function parameters were derived.

The systemic hemodynamic results are shown in Figure 4. HR, CO, and MAP were statistically higher for the RBC-DOX group than for the free DOX group. On the other hand, SVR was higher for the free DOX group than for the RBC-DOX group. HR, MAP, CO, and SVR values of the RBC-DOX group approximated the control group values more closely than the free DOX group values. A similar trend is observed in the cardiac function parameters (Figure 5(A)), where all parameters related to the heart’s ability to pump blood, namely SV, EF, SW and dP/dt, were statistically larger, and closer to the control group, for the RBC-DOX group than for the free DOX group. The LVSP was also scientifically higher for the RBC-DOX compared to the free DOX group. The LVDP was statistically lower, and also closer to the control group, for the RBC-DOX group relative to the free DOX group. The PV loops from which the functional parameters were derived are shown in Figure 5(B). These results suggest increased cardiac performance for the RBC-DOX group as compared to the free DOX group in all measured hemodynamic and functional parameters.

## Discussion

The principal finding of this study is that RBC-DOX has superior anticancer activities and decreased toxicity compared to equivalent doses of free DOX in a xenograft mouse model of human colorectal adenocarcinoma. The increased anticancer effects of RBC-DOX were demonstrated in the *in vitro* HT-29 cell culture experiments and the extended pharmacological effects induced by the encapsulation of DOX in the RBCs. This study shows several important points: (a) RBC-DOX was readily taken up by colon cancer cells and displayed prolonged intravascular retention; (b) encapsulation of DOX in RBC resulted in a decreased distribution of the drug to the principal sites of acute and chronic toxicity of free DOX, namely the heart and the skin, as well as markedly lowered cardiotoxicity and myelosuppression; (c) RBC-DOX displayed an enhanced therapeutic efficacy in a xenograft mouse model of human colorectal adenocarcinoma.

The lower IC_50_ and the lower percent survival at the maximum concentration of 3 µg/mL for the RBC-DOX, as compared to free DOX, are indicative of increased tumor cytotoxicity and potency for the RBC-DOX. *In vivo*, the increased antitumorigenic effect was demonstrated by a significantly smaller tumor size for the RBC-DOX treated group, compared to the free DOX group. This increased antitumor cytotoxicity can be attributed to the liposomal nature of RBCs. It has been shown extensively that using spherical lipid vesicles, namely liposomes, as drug carriers for chemotherapeutic agents increases the agent’s antitumorigenic effects (Abraham et al., 2005; Niu et al., 2010; Yingchoncharoen et al., 2016). This effect has been attributed, both *in vitro* and *in vivo*, to the increased permeability of the enclosed drug to the cellular lipid bilayer, allowing increased drug uptake by cancerous cells. This effect, however, often involves a drug enclosing liposome that is at least an order of magnitude smaller than the enclosing cell. Therefore, when using RBCs, more complex cell-cell interactions are likely to be taking place. Another complementary effect of enclosing DOX in the RBCs is that the diffusion of the drug is now also limited by the diffusion of the RBCs. This generates higher effective concentrations of DOX in the regions surrounding clusters of RBC-DOX. *In vitro* this allows the drug to be contained in concentrated regions, potentially accumulating near the tumor cells and leading to the observed increased antitumor activity and cytotoxicity. This effect also proves especially relevant *in vivo,* since the complex vasculature of tumors are ideal environments for these clusters to form as the liposomes extravasate to the peritumor region (Yingchoncharoen et al., 2016). However, the larger size of RBCs as compared to traditional liposomes might limit this effect, which might explain why there was not a statistically higher concentration of DOX in the tumor of the RBC-DOX group as compared to the free DOX group (Figure 2(C)). Nevertheless, the size of the tumor in the RBC-DOX group was significantly smaller than the size of the tumor in the free DOX group, potentially due to the extended bioavailability of DOX from the RBC-DOX.

The liposomal nature of the RBCs also plays a role in maintaining an increased long term effective concentration of DOX in the body as demonstrated by the pharmacokinetics (Figure 1). Decreased drug clearance from the body, which is characteristic of liposomes, is often attributed to decreased immune and renal filtration of the enclosed drug (Yingchoncharoen et al., 2016). In RBCs, the inherent biomimetic ability of the membrane allows for an even superior avoidance of undesired immune responses or enzymolysis (Muzykantov, 2010; Xu et al., 2016; Sun et al., 2017). Extended bioavailability combined with the clustering effects mentioned before, increased the probability for the drug to arrive to the peritumor area (Gabizon et al., 2003; Urva et al., 2009). Since no significant differences in tumor DOX concentration were found between free DOX and RBC-DOX, it is likely that RBC-DOX resulted in repeated exposures at equivalent concentrations of DOX over longer periods of time due to increased bioavailability. The repeated exposure, resulting from a recurring flow of RBC-DOX through the tumor vasculature led to a significant decrease in tumor size without a significant increase in DOX concentration for the RBC-DOX group. With RBCs as the carrier, the clearance of RBC-DOX appears to involve the spleen and the liver. This is supported by the DOX biodistribution results, where statistically higher DOX concentrations of about twice as much in the liver and three times as much in the spleen were found for the RBC-DOX group compared to the free DOX group. The lungs also had a significantly larger concentration of DOX for the RBC-DOX group. The concentration of large liposomes (5 − 10 µm) in the lungs has been observed previously, and it is attributed to the effect of bloodborne macrophages, which tend to engulf the liposomes and then migrate to the alveoli where they become resident alveolar macrophages (Poste et al., 1982).

The myelosuppression results also present interesting findings. For relative white cell in general and lymphocytes, neutrophils, and eosinophils, the cell count was higher for the RBC-DOX group than the DOX group, but still lower than the control group. This suggests that a certain degree of myelosuppression is still present with the usage of RBC-DOX, however, not as significant as that present under free DOX. Interestingly, the monocyte cell count for the RBC-DOX group is higher than that of both the DOX and control groups. This serves as evidence of a functioning immune system, suggesting that the RBC-DOX induced myelosuppression is not enough to suppress the immune response. On the other hand, this is also evidence in favor of an immune foreign body response to the RBC-DOX. Liposomes have been shown to cause an immune response when used as drug delivery alternatives (La-Beck & Gabizon, 2017). Specifically, pegylated doxorubicin-loaded liposomes can lead to blood complement immune response activation upon first exposure, leading to hypersensitivity (Chanan-Khan et al., 2003). These types of liposomal induced immune responses were one of the motivations to use RBCs as a drug carrier alternative. However, RBCs themselves are intricately related to the action of immune system, specifically monocytes (de Back et al., 2014). The mononuclear phagocyte system (MPS), is comprised of monocytes and macrophages in the blood, spleen, and liver, which often work towards clearing damaged red blood cells (Chanan-Khan et al., 2003; de Back et al., 2014; La-Beck & Gabizon, 2017). In this process, cells that are unable to deform through the endothelial slits of the spleen will then be cleared out by resident macrophages. Therefore the size, stability, and mechanical properties of the RBC-DOX liposomes is highly relevant in order to prevent splenic filtering. The process of RBC loading with DOX via electroporation is likely to induce changes in the size, stability, and mechanical properties of the loaded RBCs. However, our previous studies with RBCs loaded with different agents via electroporation did not detect any significant loss in hemoglobin or increased RBC sequestration after large volume exchange transfusion relative to the low dose/volume of RBC-DOX used in this study (Cabrales et al., 2008; Villela et al., 2009). Previous studies have also reported the changes in cell volume, rheology, and deformability of RBCs loaded with different agents via electroporation. Results indicate that the process of electroporation to load drugs into RBCs does not significantly affect their mean corpuscular volume, blood viscosity, or change their deformability (Cabrales et al., 2008; Villela et al., 2009). Lastly, the *in vivo* stability of RBC-DOX in the circulation was confirmed by the pharmacokinetics of RBC-DOX obtained in this study, where RBC-DOX presented a half-life of over 3 h, suggesting that RBCs loaded with DOX via electroporation can circulate for long periods of time without being trapped in the spleen and liver. The RBC-DOX’s half-life as well as the evidence from previous studies showing that minimal changes in the loaded RBCs’ structural properties result from the electroporation process, suggest that the accumulation of DOX in the spleen of RBC-DOX treated animals might be a result of a change in deformability towards the end of the RBC-DOX vesicle lifecycle. As the DOX is released from the RBC vesicle, the corpuscular volume and stiffness might change leading to eventual filtering by the spleen. Even small changes in deformability or changes in RBC surface can be detected by the splenic endothelial slits. This can lead to an upregulation in the number of splenic macrophages as a response to an increased number of cells being filtered out in the spleen. The loading process might have also caused the loss of some important surface markers in the RBCs. The absence of some of these markers such as CD47 might trigger a phagocytic response against the RBC which now will be treated as a foreign body, effectively causing an increase in circulating monocytes in order to elicit the response (Oldenborg et al., 2000; de Back et al., 2014).

The systemic hemodynamics and cardiac function results are all suggestive of decreased cardiac toxicity when using RBC-DOX as compared to free DOX. The elevated HR, MAP, CO, SV, EF, SW, and contractility (Figures 4,5) of the RBC-DOX group, as compared to the DOX group, serve as direct evidence of a more stable cardiovascular performance. The increased systemic vascular resistance of the free DOX group is suggestive of the presence of vasoconstriction. This vasoconstriction can be traced back to the effects of endothelin-1, which has been previously shown to increase in doxorubicin-treated mice (Bien et al., 2007). Increases in endothelin-1 are also associated with increases in the calcium load of cardiomyocytes, eventually leading to apoptosis and subsequent cardiac dysfunction (Mitry & Edwards, 2016). Based on this hypothesis, the decreased SVR of the RBC-DOX group might be indicative of a decreased upregulation of endothelin-1, and therefore, decreased cardiac damage associated with it. Furthermore, endothelin-1 is also associated with increased myocardial stiffness, which is in itself directly related to the cardiac dysfunction. This effect can be observed in the increased LVDP, which is significantly more elevated in the DOX group, than in the RBC-DOX group. According to Laplace’s law, elevated pressure suggests elevated wall tension, and elevated wall tension in diastole is often due to increased stiffness. This is further evidence in favor of decreased cardiac toxicity when using RBC-DOX as compared to free DOX. As a potential explanation of why RBC-DOX leads to improved cardiac function relative to free DOX one can look at the biodistribution of DOX in the heart (Figure 2(C)). For the RBC-DOX group, the DOX concentration in the heart was almost half of the concentration found in the heart for the free DOX group. The constraining of DOX inside the RBC makes it harder for it to reach the cardiac tissue when flowing in the highly dynamic ventricular and aortic flow profiles, as the DOX would not only have to diffuse from the circulation into the tissue, but also through the RBC membrane. Furthermore, the likely alterations in RBC membrane stiffness for RBC-DOX are likely to decrease the ability for these modified cells to reach the coronary circulation from the aortic arch, since the behavior in turbulent flow, for these modified cells, will be different than the behavior of regular cells. Further studies in this area are required for a comprehensive understanding of what is the mechanism for decreased cardiac toxicity.

In most cases, cancer chemotherapy is limited by the low therapeutic index of the anticancer drugs due to serious toxicity to normal tissues. Indeed, the therapy-limiting toxicity of DOX is cardiomyopathy, which may lead to congestive heart failure and death (Swain et al., 2003). The goal of loading RBCs with DOX was to improve the biodistribution characteristics and changes the toxicologic properties of DOX. These studies demonstrated that the accumulation of DOX from RBC DOX in the skin and heart were significantly lower than that of free DOX. Tissue levels of DOX were reduced by RBC-DOX delivery, the decreased concentration of DOX at these sites is likely to result in a reduced risk of the development of side effects. Indeed, the biochemical and hematological analyses demonstrated that the improved therapeutic efficacy of RBC-DOX was obtained without an increase in toxicity to the heart or to the bone marrow. As for the indicators of myelosuppression, the higher level of total white cell numbers, lymphocytes, and monocytes in the RBC-DOX group indicated that RBC-DOX decreased the toxicity of myelosuppression of the encapsulated drug. The increased accumulation of DOX from RBC-DOX in the liver, spleen, and lung compared to the free DOX may be related to preferential accumulation of DOX-loaded RBCs in organs of the reticuloendothelial system.

Limitations of this study include the need for a more comprehensive understanding of what is happening in the vascular region near the tumor, as this might elucidate the mechanism through which RBC-DOX achieves increased antitumorigenic effects. Current studies by our group are focused on using fluorescently labeled DOX, and RBC-DOX for visualization in window chamber model implanted tumors. This will allow to understand the diffusive behavior of DOX and RBC-DOX as well as verify observation made in this initial study. Future studies should also focus on determining the changes in the RBC membrane stiffness induced by the loading procedure specifically for DOX. This will allow to determine whether changes in RBC membrane stiffness might be responsible for the observed biodistribution of the RBC-DOX. Although, no obvious alteration in spleen function was observed in the animals treated with RBC-DOX, the acute and long-term effects of the high DOX accumulation in the spleen should be explored in future studies. The most sensitive animal models for each study were used, as it is impossible to perform all the studies in the same set of animals. Tumorless mice (C57BL) were used for short term pharmacokinetics studies, as they are the most frequently used mice strain for pharmacology. Immunodeficient nude mice were used for anti-tumor evaluation, because they allow for the evaluation of xenograft tumors, although they were not used for pharmacokinetics and cardiotoxicity studies because they do not represent normal human physiology due to their inhibited immune system. BALB/C were used for cardiotoxicity studies as they can tolerate higher cumulative doses of DOX with a well-defined phenotype of DOX-induced cardiotoxicity, including decreased cardiac systolic function, ventricular enlargement, and other similar histological alterations to those occurring in humans exposed to DOX. Future studies with RBC-DOX should include a side-by-side comparison to clinically relevant current ways to administer DOX, in the form of pegylated liposomal doxorubicin, Doxil®, including the aspects measured in the current study and evaluation of their removal pathways. Clinical results indicate that Doxil® is cardiotoxic with increasing cumulative dose, and liposomes like Doxil® and other particulate delivery systems, depend on the reticuloendothelial system for removal. Doxil® clearance is mediated by the fixed macrophages of the liver, spleen, and lungs that constitute the reticuloendothelial system and is assisted by opsonins that facilitate macrophage uptake of foreign particulates, whereas RBC are removed by liver macrophages (Kupffer cells) or captured and metabolized in the spleen and spleen. Therefore, comparison on the implication of their removal process should be studied carefully in the future.

## Conclusion

This study successfully demonstrated a simple procedure for loading DOX into RBCs. It also demonstrated that RBC-DOX has increased antitumorigenic effects, and decreased myelosuppression and cardiac toxicity when compared to equivalent administrations of free DOX. These results should encourage the further development of studies to better understand the potential long-term effects of this line of treatment, as well as ways to efficiently translate this methodology into a clinically relevant setting.

## Supplementary Material

RBC_DOX_1_4_19_supplemental.docx
