# Supplementary material for: Doxorubicin-loaded red blood cells reduced cardiac toxicity and preserved anticancer activity
Source: Drug Deliv. 2019 Mar 31;26(1):433–42. doi: 10.1080/10717544.2019.1591544 (PMC6450495; doi:10.1080/10717544.2019.1591544)
Supplement: RBC_DOX_1_4_19_supplemental.docx [file IDRD_A_1591544_SM5649.docx]

# **Supplemental Figure 1**

# ****

# **Supplemental Figure 2**

# **Supplemental Figure Captions**

**Supplemental Figure 1:** Loading efficiency for RBC-DOX. Red line represents the best fit line between data points. Dotted red lines represent the 95% confidence interval of the fit.

**Supplemental Figure 2:** *In vitro* cytotoxicity in HT-29 cells as represented by the percent survival as a function of the concentration of the substance. The DOX curve is shown in red and the RBC-DOX curve is shown in blue. Dotted black line represents the *IC*_50_ value.
